# Ocular wavefront aberrometry as an adjunct in surgical decision-making for pediatric posterior polar cataract with posterior lenticonus: a case report

**DOI:** 10.3389/fmed.2026.1911201

**Published:** 2026-09-14

**Authors:** Yu Zhang, Yanyu Shen, Zhe Zhang

**Affiliations:** 1 Shenzhen Eye Hospital, Shenzhen Eye Medical Center, Southern Medical University, Shenzhen, China; 2 State Key Laboratory of Ophthalmology, Zhongshan Ophthalmic Center, Sun Yat-sen University, Guangdong Provincial Key Laboratory of Ophthalmology and Visual Science, Guangzhou, China

**Keywords:** case report, dysfunctional lens index (DLI), ocular wavefront aberrometry, pediatric cataract, posterior lenticonus

## Abstract

This report presents a 5-year-old boy with unilateral congenital cataract and posterior lenticonus, where wavefront aberrometry objectively quantified lenticular aberrations to resolve surgical timing uncertainty. Despite 0.3 corrected visual acuity and paracentral opacity, iTrace analysis revealed severe intraocular aberrations (high-order aberrations = 1.426 μm, dysfunctional lens index = 0.34,) uncorrectable with spectacles or mydriasis. One month following phacoemulsification with primary IOL implantation, the best-corrected visual acuity (BCVA) improved from 0.3 to 0.8. Aberrometry provides critical functional data to supplement morphological assessment in borderline pediatric cataracts, optimizing developmental visual outcomes.

## Introduction

Pediatric cataract management requires balancing amblyogenic risks against surgical complications. While central opacities >3 mm warrant prompt intervention, peripheral cataracts present therapeutic dilemmas. Current decisions rely on visual acuity and slit-lamp findings, often overlooking optical quality from lenticular aberrations. We demonstrate how wavefront aberrometry quantified functional impairment in a borderline case, justifying earlier surgery than traditionally indicated.

## Case presentation

A 5-year-3-month-old boy presented with 2-year history of right eye (RE) leukocoria. Multiple centers diagnosed RE congenital cataract but deferred surgery due to inferonasal opacity sparing the visual axis.

Uncorrected visual acuity was 0.25 in the right eye and 0.8 in the left eye. With refractive correction, best-corrected visual acuity measured 0.3 (Plano) in the right eye and 1.0 (+1.25DS −1.00DC × 5°) in the left eye. Slit-lamp examination revealed lens opacity in the inferonasal quadrant of the right eye (Figure 1). Fundus photography demonstrated partial obscuration of the temporal parafoveal area in the macula (Supplementary Figure 1). Preoperative swept-source optical coherence tomography (SS-OCT) and B-scan ultrasonography confirmed posterior lenticonus with localized posterior capsule thinning and posterior ectasia, though no frank capsular defect was identified. Ancillary tests including optical coherence tomography (OCT) and specular microscopy showed no abnormalities. Final diagnoses: (1) Congenital cataract in the right eye; (2) Posterior lenticonus of the right crystalline lens.

**Figure 1:**
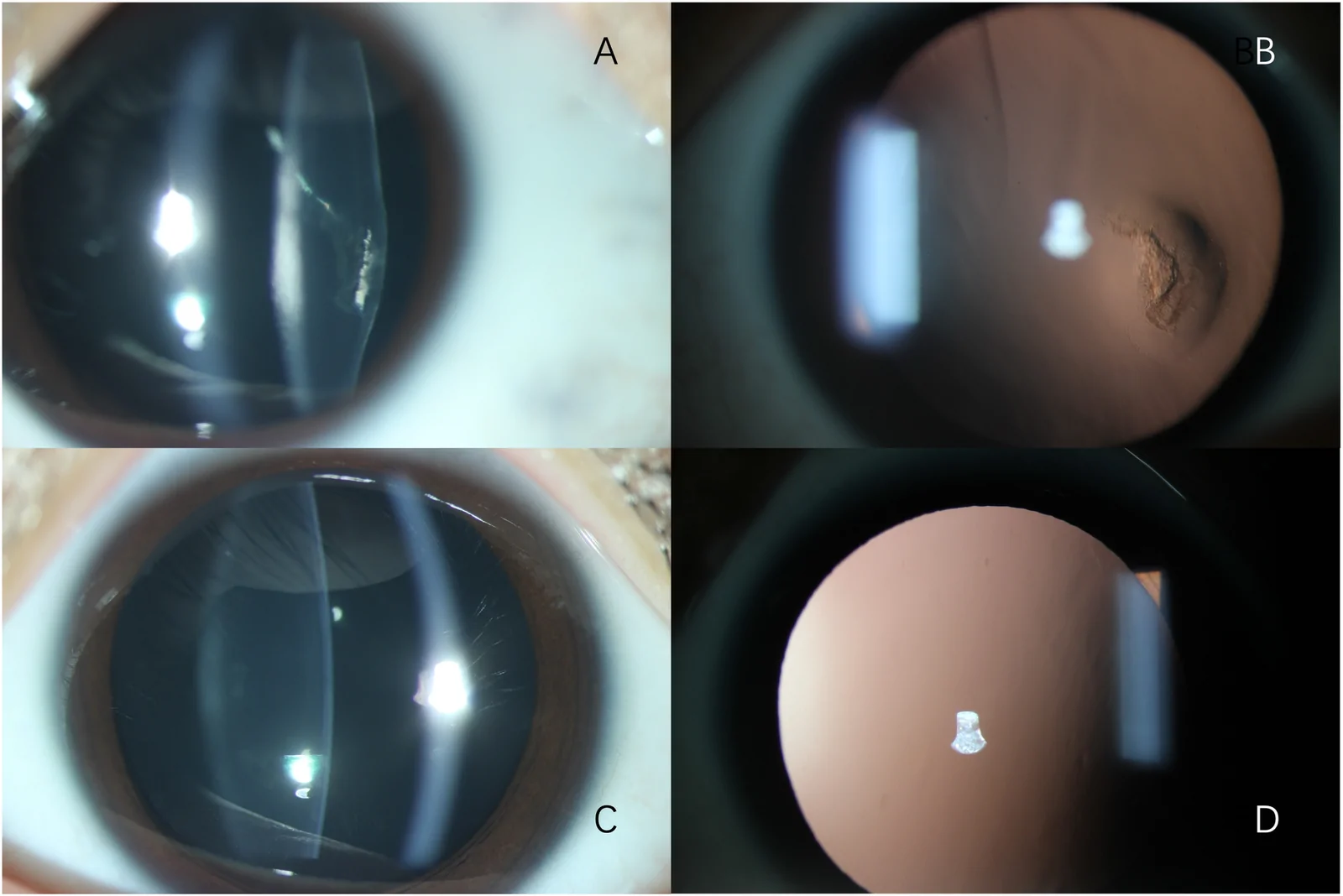
Slit-lamp examination revealed lens opacity in the inferonasal quadrant of the right eye **(A,B)**, slit-lamp photograph of the left eye **(C,D)**.

## Diagnostic aberrometry

The iTrace aberrometer (Tracey Technologies, USA) was performed under pharmacologic mydriasis in standardized low-light conditions. Aberrometry analysis demonstrated significant internal aberrations in the right eye, with a root mean square (RMS) defocus error of +1.734 μm through a 3.0 mm pupil—indicating myopia. Astigmatism measured 2.507 μm at axis 54°. Total higher-order aberrations (HOA) reached 1.426 μm, comprising: coma 1.022 μm@331°, trefoil 0.875 μm@18°, and secondary astigmatism 0.332 μm@156°. Conversely, corneal HOA was minimal (0.108 μm), with dominant lower-order aberrations (0.704 μm) primarily manifesting as astigmatism (0.703 μm@80°) (Figure 2).

**Figure 2:**
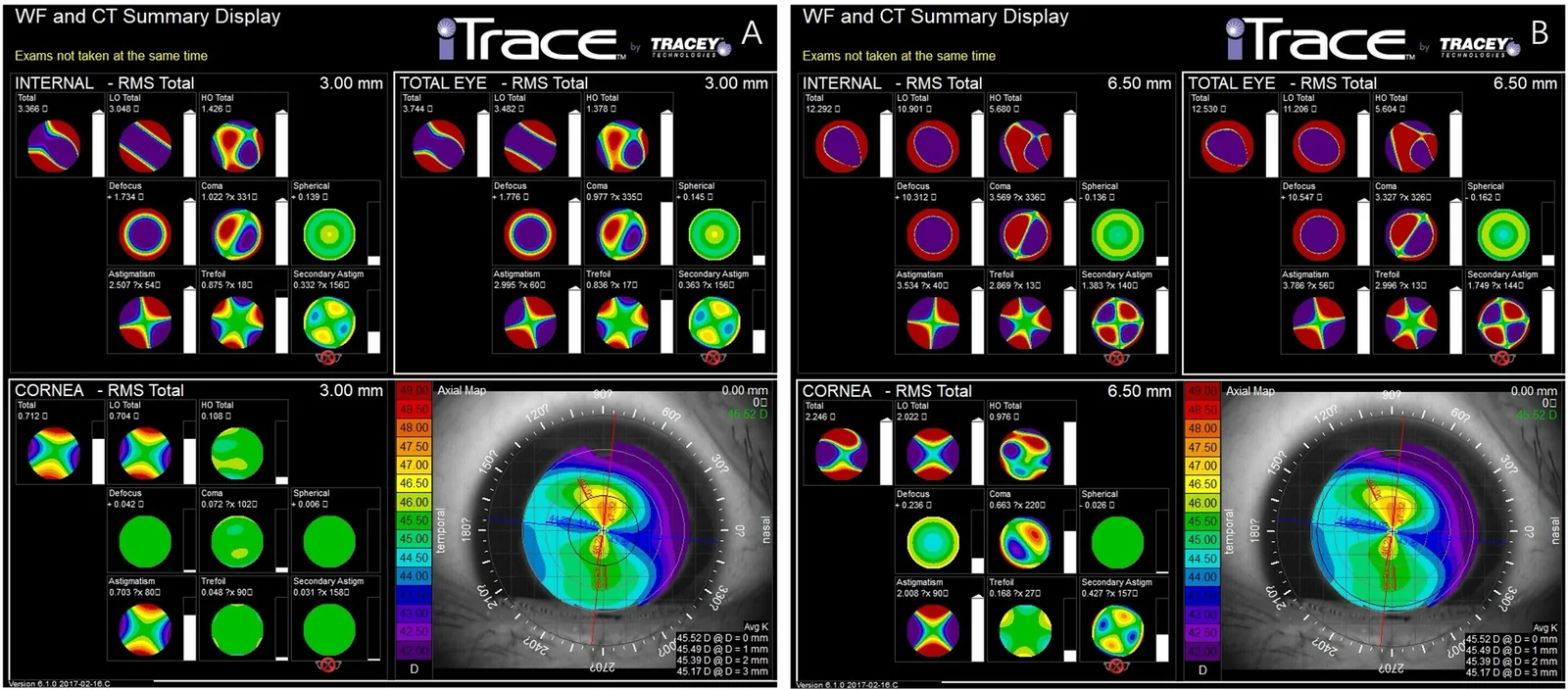
The iTrace aberration analysis displays the aberrations for 3 mm pupil zone **(A)** and 6.5 mm pupil zone **(B)**.

Alternative conservative management with pupillary dilation paradoxically increased aberrations: 6.5 mm pupil analysis revealed elevated intraocular HOA (5.680 μm), including coma 3.569 μm@336°, trefoil 2.869 μm@13°, and secondary astigmatism 1.383 μm@140° (Figure 2).

The dysfunctional lens index (DLI), quantifying intraocular optical quality (higher values indicating superior performance), was registered critically low at 0.34 in the right eye (Figure 3A)—substantially below the normative threshold of 10 (Figure 3B).

**Figure 3:**
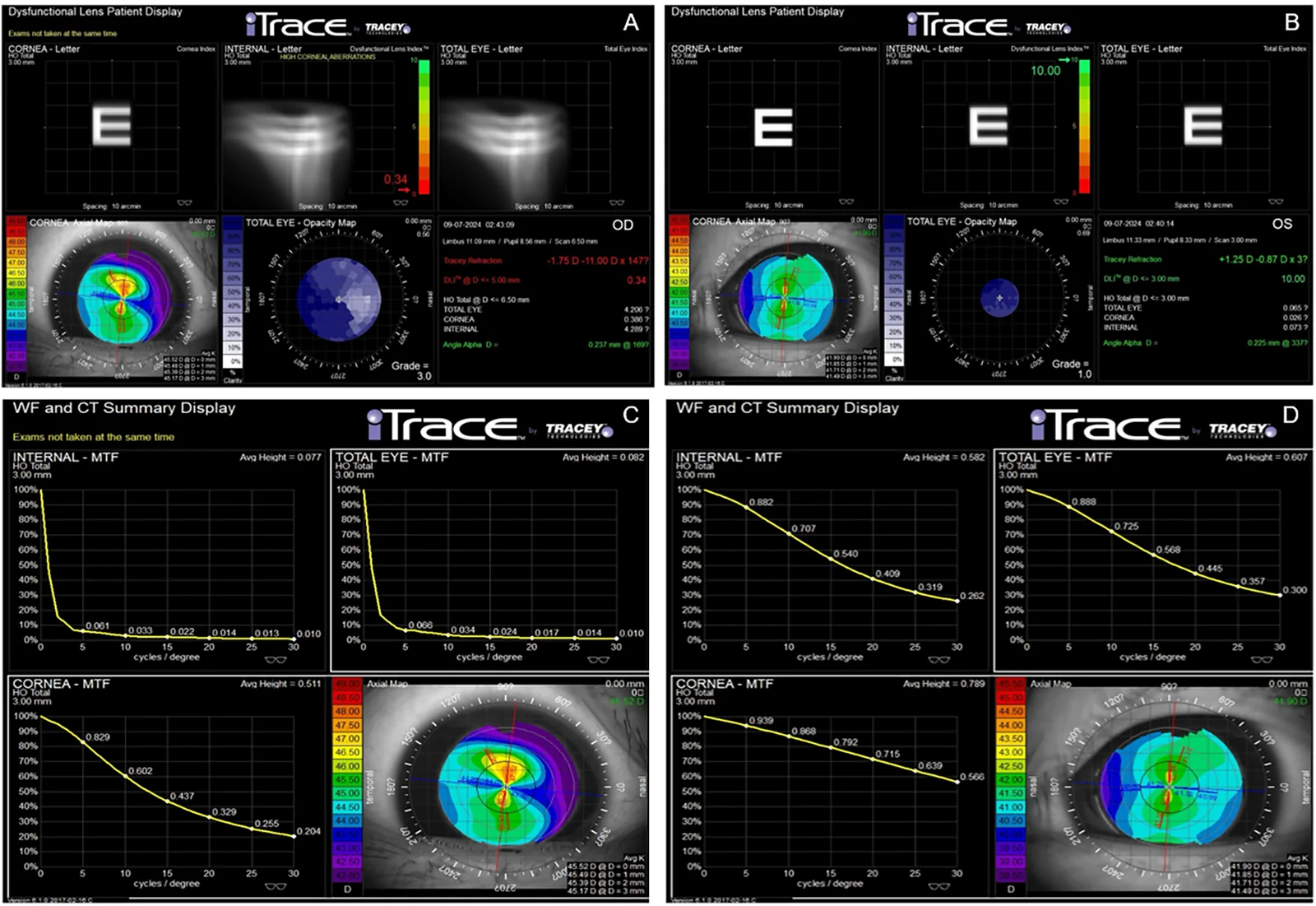
The dysfunctional Lens Index (DLI) in the right eye was critically low at 0.34 **(A)**, while the left eye showed normal values **(B)** modulation transfer function (MTF) analysis further objectified the visual impairment. At 5 cycles/degree (cpd), the right eye's internal MTF was severely depressed (0.061) compared to the healthy left eye (0.882) **(C,D)**.

Modulation transfer function (MTF) analysis further objectified the visual impairment. At 5 cycles/degree (cpd), the right eye's internal MTF was severely depressed (0.061) compared to the healthy left eye (0.882) (Figures 3C,D). Given the relatively preserved corneal MTF (0.829 at 5 cpd), the marked decline in total ocular MTF (0.066 at 5 cpd) was highly likely attributable to the diseased lens. This MTF degradation independently corroborates the decline in DLI. Together, these metrics demonstrate that profound intraocular aberrations induced by the peripheral opacity significantly impaired visual quality.

While refractive correction remains a viable option for managing focal lens opacities, this case demonstrates three clinically significant findings: (1) dominant intraocular-origin aberrations, (2) high-order aberrations non-correctable with conventional spectacles, and (3) paradoxical exacerbation of optical aberrations following pupillary dilation as conservative management, which may compromise diagnostic data quality. Notably, the 5-year-old patient presented with a best-corrected visual acuity of 0.3, placing them at a critical developmental window for amblyopia therapy intervention.

Given irreversible impairment of visual development during the critical period, cataract extraction was indicated to optimize visual rehabilitation potential.

Given the anatomical fragility of the posterior capsule in posterior lenticonus, surgical planning prioritized gentle hydrodissection and low-vacuum phacoemulsification with primary implantation of a monofocal intraocular lens (IOL) DCB00 (Abbott Medical Optics, United States) + 14.0D in the capsular bag. Primary posterior capsulotomy and anterior vitrectomy were performed to ensure a clear visual axis and prevent secondary PCO. The target postoperative refraction of +1.23D was calculated using the Barrett Universal II formula, with refractive planning methodology referencing our institutional protocol (1).

The postoperative refractive outcomes showed progressive improvement over time. At 1 week postoperatively (Figure 4), the uncorrected visual acuity (UCVA) was 0.32, while the best-corrected visual acuity (BCVA) reached 0.8 with a refraction of +2.50DS −1.00DC × 162°. By 1 month, the UCVA had improved to 0.40, and the BCVA remained stable at 0.8 with a slight refinement in refraction to +2.25DS −1.25DC × 169°.The BCVA enhancement over one month confirms significant visual rehabilitation.

**Figure 4:**
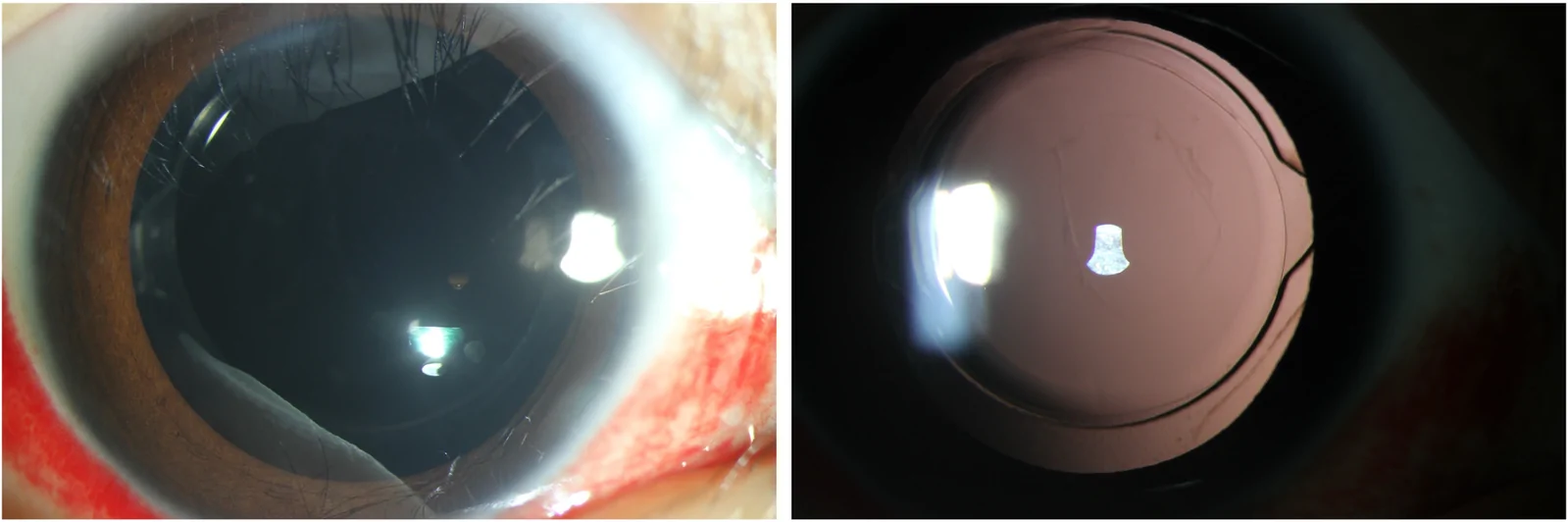
Slit-lamp photograph at 1 week postoperatively.

## Discussion

In this case, aberrometry provided useful adjunctive functional evidence of intraocular optical degradation to assist in evaluating a borderline pediatric cataract, particularly for localized opacities where traditional visual acuity metrics may underestimate functional impairment. Despite initial deferral of surgery due to sparing of the visual axis, aberrometry revealed profound intraocular optical degradation that necessitated intervention to prevent irreversible amblyopia during the critical developmental period.

The preoperative aberrometry demonstrated significant internal higher-order aberrations (HOAs), with coma (1.022 μm) and trefoil (0.875 μm) dominating the wavefront error profile. This aligns with Sachdev et al.'s findings that cortical and nuclear cataracts induce distinct HOA patterns—specifically, cortical opacities predominantly increase coma and trefoil, while nuclear opacities elevate spherical aberration (2). In this case, the inferonasal lens opacity, together with the anatomical curvature distortion from the posterior lenticonus, likely contributed to the elevated coma and trefoil aberrations, which degrade contrast sensitivity and retinal image quality independent of Snellen acuity (2, 3).

Pharmacologic mydriasis exacerbated HOAs by 300%–400% (e.g., coma increased to 3.569 μm), consistent with Applegate et al.'s demonstration that HO RMS wavefront error scales exponentially with pupil diameter (4). Their 3D model showed that for a fixed age, HOAs increase more rapidly with pupil dilation than with aging itself (4). This phenomenon explains why conservative management with pupillary dilation paradoxically degraded optical quality in this patient, a critical consideration for clinical decision-making.

The Dysfunctional Lens Index (DLI) of 0.34 objectively quantified severe lenticular optical dysfunction in this patient. Similar to the objective scatter index (OSI) in Optical Quality Analysis System (OQAS) studies, DLI serves as a sensitive biomarker for cataract-induced light scattering and aberrations (5). Hwang et al. demonstrated that OSI outperforms visual acuity in discriminating cataract severity (5), while Chen et al. emphasized that even small posterior opacities can impair visual function due to their proximity to the macula (6). In this case, the markedly depressed DLI aligned with the patient's amblyopia risk (BCVA 0.3), providing valuable adjunctive evidence of functional optical impairment. However, because DLI was primarily developed and validated in adult populations undergoing age-related cataract evaluation, standard adult normative thresholds (e.g., DLI = 10) cannot be directly extrapolated to pediatric eyes or structural anomalies such as posterior lenticonus (7). Therefore, while the low DLI value helped confirm significant lenticular dysfunction, pediatric DLI metrics should be interpreted with caution as standardized diagnostic thresholds for pediatric cataracts remain to be established.

Surgical decision-making in pediatric cataract relies on a holistic risk–benefit assessment guided by established clinical standards, including the Chinese Expert Consensus on Perioperative Management of Pediatric Cataract (2022) and the Guidelines for Diagnosis and Treatment of Amblyopia (2021). In our patient, comprehensive preoperative evaluation revealed no surgical contraindications. At 5 years of age, the baseline BCVA of 0.3 represented established amblyopia within a critical therapeutic window. While slit-lamp morphology appeared borderline, objective aberrometry (DLI 0.34, MTF 0.061, intraocular HOA 1.426 μm) confirmed severe lenticular optical dysfunction uncorrectable by pupillary dilation, confirming the indication for intervention. Intraoperatively, standard primary posterior capsulotomy with anterior vitrectomy was performed; because the central posterior capsule was surgically excised, the risk of postoperative visual axis opacification (PCO) is minimal in this 5-year-old child. Furthermore, excellent patient cooperation enabled reliable anterior segment and gonioscopic evaluation, confirming open angles with no coexisting structural anomalies, indicating a very low risk of secondary glaucoma. Regarding post-surgical refractive shift, the ocular growth and refractive trajectory at 5 years of age are substantially more predictable than in infants operated under 2 years of age. Nevertheless, because residual refractive drift remains possible, structured long-term surveillance, regular subjective refraction updates, and compliance with amblyopia therapy remain essential to sustain visual gains. Ultimately, the favorable postoperative BCVA improvement to 0.8 cannot be attributed solely to preoperative optical quality assessment; rather, it represents the synergistic outcome of precise IOL power calculation, uneventful posterior capsulotomy and vitrectomy, accurate postoperative refractive correction, and diligent amblyopia therapy leveraging residual neural plasticity.

Studies on posterior polar cataracts emphasize that surgical outcomes depend on meticulous management of capsule integrity (3), which was achieved here without complications. The +1.23D refractive target aligned with evidence supporting slight hyperopia in pediatric IOL calculations to accommodate ocular growth.

Certain practical limitations of pediatric wavefront aberrometry must be acknowledged. Diagnostic accuracy in young children can be significantly influenced by fixation instability, limited patient cooperation, fluctuations in pupil size, accommodation, and measurement repeatability. These factors may introduce measurement noise or variability, necessitating careful clinical correlation rather than direct reliance on single diagnostic readouts.

This case illustrates the potential of wavefront-guided diagnostics—particularly intraocular HOA profiling and DLI assessment—as valuable adjunctive tools for evaluating cataract-related optical dysfunction. In pediatric patients with localized opacities, these metrics may help capture critical amblyogenic risk factors that conventional visual acuity metrics might underestimate, potentially enabling timely intervention during the plastic phase of visual development. Nevertheless, as a single case report, these observations remain strictly hypothesis-generating. Wavefront aberrometry, DLI, and MTF metrics cannot currently serve as standalone criteria for determining surgical timing in pediatric cataract. Prospective cohort studies and multicenter pediatric series are required to establish valid pediatric normative thresholds, clinical applicability, and true incremental value. Additionally, while 1-month postoperative follow-up demonstrated clear early visual rehabilitation (BCVA 0.8), longer-term longitudinal follow-up is necessary to monitor myopic shift, refractive stability, and sustained visual development.

## Declarations

This case report was prepared in accordance with the CARE (CAse REport) guidelines. Prior to surgery, comprehensive written informed consent was obtained from the patient's parents, and all relevant consent documentation was duly signed.

## Patient's perspective (written by the patient's parents)

“When the doctors showed us the simulated ‘E’ optotype images generated by the aberrometer (Figures 3A,B), we finally understood his condition. Seeing the severely distorted image made it intuitively clear to us that a lesion in his ‘internal lens’ was causing the profound blurring and visual distortion. This visual evidence gave us the confidence to consent to the surgery, despite having it deferred at other hospitals previously. Postoperatively, we are satisfied with the treatment outcomes. We have noticed significant improvements in his daily life: his walking balance is much better, and he rarely trips or falls anymore. Furthermore, he is now able to concentrate on reading for much longer periods without complaining of fatigue.”

## Conclusion

This case suggests that posterior lenticonus, even with peripheral opacity, can cause severe HOAs that compromise visual development. Aberrometry provides useful adjunctive functional data to complement morphological and visual acuity assessments in borderline pediatric cataracts, aiding patient-specific surgical decision-making. However, surgical timing should be determined through a comprehensive individualized assessment, including patient age, amblyopia risk, lens morphology, posterior capsule anatomy, surgical risk, and expected visual benefit. Findings from a single case are insufficient to support the use of these optical quality metrics as independent or decisive surgical indicators.

## Data Availability

The raw data supporting the conclusions of this article will be made available by the authors, without undue reservation.
